# Single-Agent and Fixed-Dose Combination HIV-1 Protease Inhibitor Drugs in Fission Yeast (*Schizosaccharomyces pombe*)

**DOI:** 10.3390/pathogens10070804

**Published:** 2021-06-24

**Authors:** Jiantao Zhang, Kasey Vernon, Qi Li, Zsigmond Benko, Anthony Amoroso, Mohamed Nasr, Richard Y. Zhao

**Affiliations:** 1 Department of Pathology, University of Maryland School of Medicine, Baltimore, MD 21201, USA; Jiantao.Zhang@som.umaryland.edu (J.Z.); kvernon@umaryland.edu (K.V.); Qi.Li@som.umaryland.edu (Q.L.); benko.zsigmond@science.unideb.hu (Z.B.); 2 Institute of Human Virology, University of Maryland School of Medicine, Baltimore, MD 21201, USA; aamoroso@ihv.umaryland.edu; 3 Drug Development and Clinical Sciences Branch, Division of AIDS, NIAID, National Institutes of Health, Bethesda, MD 20892, USA; mnasr@niaid.nih.gov; 4 Department of Microbiology-Immunology, University of Maryland School of Medicine, Baltimore, MD 21201, USA; 5 Institute of Global Health, University of Maryland School of Medicine, Baltimore, MD 21201, USA

**Keywords:** human immunodeficiency virus type 1 (HIV-1), protease (PR), protease inhibitor (PI), fission yeast (*Schizosaccharomyces pombe*), viral drug resistance, cell proliferation, HIV-1 PR enzymatic assay, single-agent drug, fixed-dose combination drug

## Abstract

Successful combination antiretroviral therapies (cART) eliminate active replicating HIV-1, slow down disease progression, and prolong lives. However, cART effectiveness could be compromised by the emergence of viral multidrug resistance, suggesting the need for new drug discoveries. The objective of this study was to further demonstrate the utility of the fission yeast cell-based systems that we developed previously for the discovery and testing of HIV protease (PR) inhibitors (PIs) against wild-type or multi-PI drug resistant _M11_PR that we isolated from an infected individual. All thirteen FDA-approved single-agent and fixed-dose combination HIV PI drugs were tested. The effect of these drugs on HIV PR activities was tested in pure compounds or formulation drugs. All FDA-approved PI drugs, except for a prodrug FPV, were able to suppress the wild-type PR-induced cellular and enzymatic activities. Relative drug potencies measured by EC_50_ in fission yeast were discussed in comparison with those measured in human cells. In contrast, none of the FDA-approved drugs suppressed the multi-PI drug resistant _M11_PR activities. Results of this study show that fission yeast is a reliable cell-based system for the discovery and testing of HIV PIs and further demonstrate the need for new PI drugs against viral multi-PI resistance.

## 1. Introduction

Infection of human immunodeficiency virus type 1 (HIV-1) continues to be a threat to global health. Although there is still no cure, successful treatment of HIV-infected individuals with combination antiretroviral therapies (cART) can eliminate all active replicating viruses and halt the progression of HIV disease, thus prolonging patients’ lives.

Currently, there are eight different classes of antiretroviral drugs with five targeting the virus and three targeting the host [1,2,3]. Drugs that target the virus include reverse transcriptase inhibitors such as nucleoside reverse transcriptase inhibitors (NRTIs) and non-nucleoside reverse transcriptase inhibitors (NNRTIs), protease inhibitors (PIs), integrase inhibitors (INIs), gp41 inhibitor, and gp120 inhibitor. Drugs that target the host cells are CD4 inhibitor, CCR5 inhibitor, and pharmacokinetic enhancers. Note that unlike other antiretroviral drugs, pharmacokinetic enhancers do not interfere with viral replication but rather boost the drug efficacy by inhibiting host cytochrome P450 (CYPs) enzymes [1,3]. So far, there are a total of 58 US Food and Drug Administration (FDA)-approved therapeutics with 45 single-agent antiretroviral drugs and 13 fixed-dose combination drugs that typically comprise two or more antiretroviral agents. The most recent approved single-agent drug is Fostemsavir (Rukobia, ViiV Healthcare), which is a gp120 attachment inhibitor and the first-in-class drug [2]. It prevents HIV infection by blocking gp120 from infecting CD4-positive cells. This drug covers people who are failing cART due to multidrug resistance. Combination drugs are used to minimize the emergence of drug resistance, improve drug efficacy, or prolong drug effect. The most recent example of an FDA-approved combination antiretroviral drug regimen is Cabenuva, which combines an INI Cabotegravir with a NNRTI Rilpivirine. It only requires once a month injection rather than having to take an oral dose every day [2]. The most successful therapeutic strategy at present is the use of cART, which consists of three to four antiretroviral drugs of different classes. Among them, HIV-1 PI drugs are one of the most potent classes of antiretroviral drugs, because HIV-1 protease (PR) plays an essential role in viral replication and reproduction [4,5,6,7].

HIV-1 PR is a dimeric aspartyl protease that consists of two identical subunits with 99 amino acids, which specifically cleaves the viral polyprotein precursors into mature enzymes and structural proteins [4,8,9]. This proteolytic activity is essential for the production of mature and infectious viral particles. Therefore, HIV-1 PR is a pivotal target for therapeutic intervention. Indeed, PR has been an important therapeutic target, and its use ushered in the era of highly active antiretroviral therapy when PI drugs were used in combination with other classes of antiretroviral drugs [10,11].

Most of the FDA-approved PI drugs are competitive inhibitors against the active enzymatic site of HIV-1 PR. Based on the nature and its inhibitory mechanism, they can be classified into two different generations [8,12]. The first-generation PI drugs are small peptidyl-based drugs that were designed to mimic the natural HIV-1 PR substrates by competitive fitting to the active enzymatic site [13]. They were based on hydroxyethylene and hydroxyethylamine isosteres. The central hydroxyl group mimics the transition state of the hydrolysis step by binding with the catalytic aspartic acid residues [8]. The second-generation PI drugs are non-peptidyl P2 ligand-based small molecule drugs designed to interrupt dimerization of HIV-1 PR and increase the genetic barrier to viral drug resistance [8]. The rationale behind the P2-ligand drug was that if a P2 ligand binds to the S2/S2′ backbone atoms of HIV-1 PR with the maximum hydrogen-bonding affinity, it will prevent the PR from accessing its nature viral substrates, such as the MA↓CA that we used in this study, thus inhibiting HIV-1 PR. In addition, high P2–S2 binding affinity makes it more tolerable to viral *PR* gene mutations thus increasing the genetic barrier to the development of viral drug resistance [14,15]. The last approved second-generation PI drug approved by FDA in 2006 was Darunavir (DRV, Prezista™). Indeed, DRV does inhibit viral drug resistant HIV-1 PRs even when most of the PI drug failed [16].

Currently, there are 13 FDA-approved PI drugs along with several combination drugs that are used in cART. For example, in the lopinavir–ritonavir (LPV/r) combination, ritonavir (RTV) serves as a booster, because a low dose of RTV inhibits cytochrome CYP3A4 enzyme-mediated LPV metabolism and the P-glycoprotein efflux pump, thereby providing effective LPV plasma exposure [17]. Besides serving as a pharmacokinetic enhancer, another PI drug Fosamprenavir (FPV) is used as a prodrug for amprenavir (APV), because FPV is metabolized by host cellular enzymes to form APV, which is the active agent. The transition from a prodrug FPV to active drug APV in the human body increases the duration of APV, making FPV a slow-release version of APV and thus prolonging the duration of the drug effect [18].

Although cART has dramatically reduced the mortality of HIV-infected patients, its effectiveness is often compromised due to the emergence of viral drug resistance, especially in the developing world [19,20,21,22]. Viral multidrug resistance is one of the main threats to the success of cART [23,24,25]. At present, more than half of the adults in Central Africa carry viral multidrug resistant viruses with an average of 10.6% throughout Africa [22]. Viral multidrug resistance was also found in pediatric patients globally [26]. Thus, constant effort is required to fight viral multidrug resistance. Like other antiretroviral drugs, viral multi-PI resistance to HIV-1 PIs is often derived from non-compliance of drug intake or prolonged PI-based cART use in the presence of viral replication. Prolonged exposure to the same PI drugs of an infected individual could enable the HIV-1 *PR*-encoding nucleotide sequence to mutate, leading to the emergence of viral drug resistance to PI drugs [27,28,29,30]. For example, a single I84V mutation could result in cross-viral drug resistance to FPV, Indinavir (IDV), Atazanavir (AZV), and Tipranavir (TPV) [19,20,21,25,31]. Consequently, none of these PIs are effective to combat viral drug resistant HIV PRs. Our laboratory isolated two multi-PI resistant HIV PRs from HIV-infected individuals that contained ten or eleven *PR* gene mutations (_M10_PR or _M11_PR), respectively [32]. Phenotypic prediction based on megadata analysis by a FDA-approved and clinical HIV-1 genotyping ViroSeq test indicated that these two HIV-1 mutant PRs might be resistant to all current FDA-approved PI drugs [32]. Therefore, viral drugs resistant to HIV-1 PI drugs do emerge in today’s patient care in the United States, including those resistant to DRV [32,33,34,35]. Hence, there is a continued need to discover new and more potent antiretroviral PI drugs to combat HIV multidrug resistance.

To facilitate the new drug discovery and testing of HIV-1 PI drugs, we developed several fission yeast (*Schizosaccharomyces pombe*) cell-based systems that allow large-scale or high-throughput drug screening against the wild-type or multi-PI drug resistant HIV-1 PRs [25,32,36]. The objective of this study was to further demonstrate the utility of those developed fission yeast cell-based systems by testing all the FDA-approved HIV-1 PI drugs that were available to us. In addition, we wanted to quantify the drug potencies in fission yeast cells by the measurement of half maximal effective concentration (EC_50_), and further compared them in pure compounds, formulated single-agent drugs, and combination drugs mixed with a booster of human cells.

## 2. Results

### 2.1. Effect of FDA-Approved Protease Inhibitor Drugs on HIV-1 Protease Measured by Colony Formation of Fission Yeast Cells

**Figure 1:**
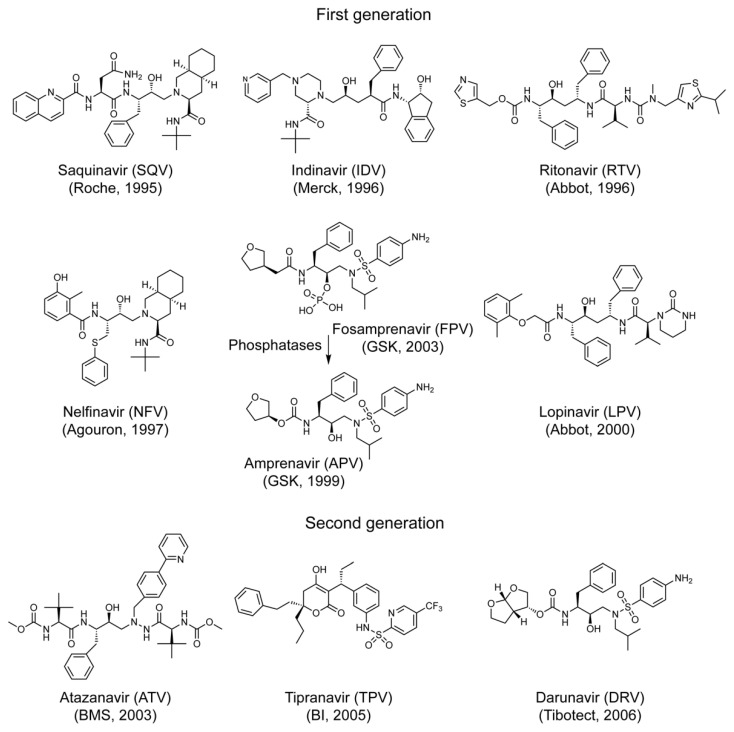
Chemical structures of the FDA-approved HIV-1 PI drugs that are used in this study. Detailed information of each drug is summarized in Table 1. The chemical structure of each protease inhibitor drug was drawn using ChemDraw software (PerkinElmer, Akron, OH, USA).

The production of HIV-1 PR protein in fission yeast inhibits cell proliferation and causes cell death that can be measured by the inability to form yeast colonies on the agar plate [25]. The goal of the experiment described here was to first test the effect of FDA-approved PI drugs on the fission yeast colony formation in the presence of HIV-1 wild type PR. The expression of HIV-1 *PR* gene is under the control of a fission yeast *nmt1* (no message in thiamine 1) promoter [37,38], i.e., the HIV-1 PR protein is only produced after thiamine is removed from the minimal growth medium for at least 16 h post-gene induction [25]. A total of thirteen FDA-approved single-agent and fixed-dose combination PI drugs were tested in this study (Table 1). Among them, seven drugs represent the first-generation of PIs, including Saquinavir (SQV), Indinavir (IDV), Ritonavir (RTV), Nelfinavir (NFV), Amprenavir (APV), Fosamprenavir (FPV), and Lopinavir (LPV). The other three drugs represent the second-generation PIs including Atazanavir (ATV), Tipranavir (TPV), and Darunavir (DRV), and three combination drugs Lopinavir/Ritonavir (LPV/r), Atazanavir/Cobicistat (COBI) (ATV/co), and Darunavir/COBI (DRV/co). The specific chemical structures of these PI drugs along with the year when these drugs were approved by FDA are shown in Figure 1. Other details are summarized in Table 1.

**Figure 2:**
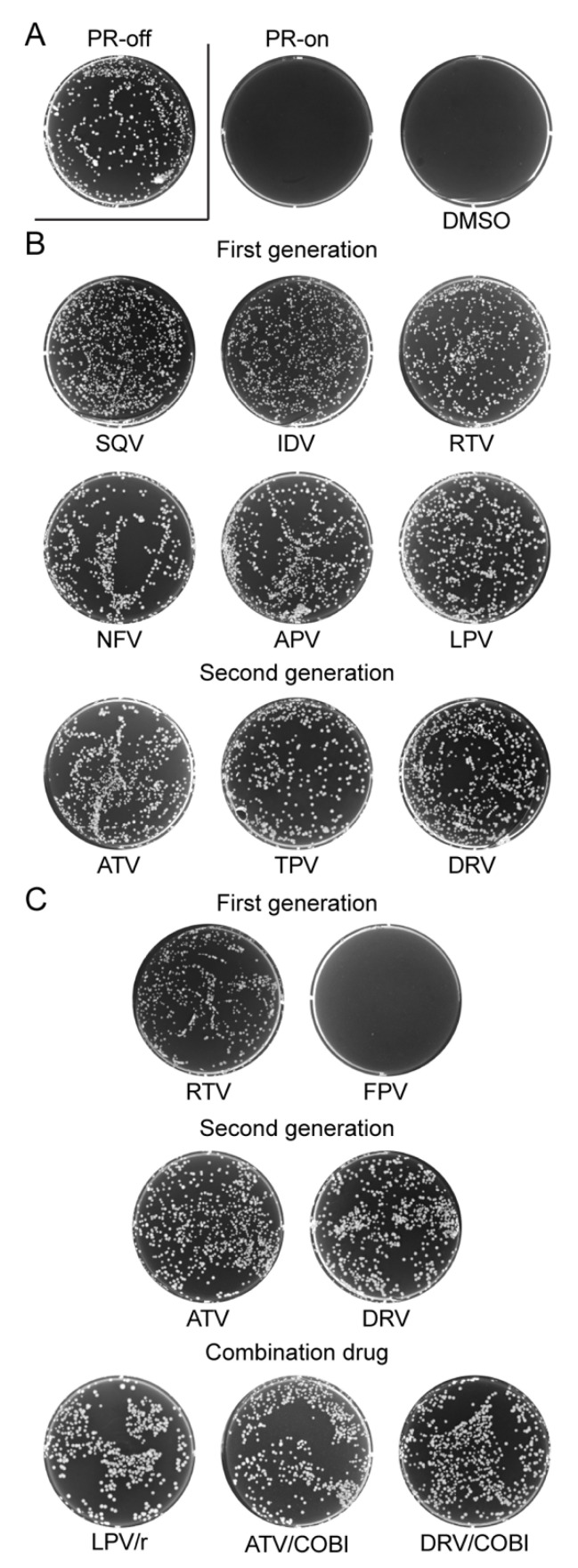
Suppression of the inhibitory effect of HIV-1 PR on fission yeast colony formation by FDA-approved PI drugs. (**A**) Assay controls: PR-off, no HIV-1 *PR* gene expression. This is the only well in this figure that HIV-1 *PR* gene was suppressed. All others are HIV-1 PR-producing wells (PR-on); DMSO, DMSO was added to the PR-on well as a mock control because it was used as a solvent to dissolve the PI drugs tested. (**B**) All single-agent PI drugs tested here were in pure chemical forms. (**C**) All single-agent PI drugs tested here were extracted from commercially available and formulated drug pills. Inducible expression of HIV-1 *PR* in fission yeast RE294 strain was achieved by removing thiamine from the growth PMG minimal medium (PR-on). No HIV-1 *PR* was expressed when 20 μM of thiamine was present in the PMG medium (PR-off) [25]. A total of 150 μM of each drug was added to the 6-well culture plate containing thiamine-free PMG agar. A total of 1 × 10^3^ RE294 PR-producing cells in the volume of 50 μL were evenly spread onto the surface of the agar culture well using a plastic rod. The 6-well agar culture plates were incubated in the 30 °C incubator. Pictures were taken at 4–5 days after incubation. Data presented represent results of three different experiments.

The effect of the above-mentioned FDA-approved PI drugs on fission yeast colony formation was tested in the presence and absence of HIV-1 PR. The results are shown in Figure 2. Specifically, a fission yeast RE294 strain, which carries a single and integrated copy of HIV-1 *PR* gene in the yeast chromosome [25,36], was used in this experiment. One thousand active growing and log-phase RE294 cells were evenly spread and cultured on the six-well agar wells containing the minimal and selective PMG medium in presence of 20 µM of thiamine (PR-off) or absence of thiamine (PR-on) with incubation at 30 °C for 4–5 days. The specific inhibitory effect of HIV-1 PR protein production on fission yeast colony formation was demonstrated as a control by showing the lack of colonies on the PR-on agar well vs. full normal-size colonies formed on the PR-off agar well (Figure 2A). To test the effect of a PI drug on the colony formation, 150 μM of each drug was added to the respective agar well in the presence of HIV-1 PR. Since all those drugs were dissolved in dimethyl sulfoxide (DMSO), to test the potential DMSO effect on the yeast colony formation, DMSO in the final concentration of 1% was added to the PR-on well as a mock control (Figure 2A). To evaluate whether other chemical ingredients in the formulation drug affect the PI activity, in this experiment, we tested the PI drugs in both pure chemical forms (Figure 2B) and in commercially available and formulated drug pills (Figure 2C).

As the result, DMSO by itself did not have any effect on fission yeast colony formation, as there were no visible colonies seen on the PR-on plate (Figure 2A). In contrast, all PI-containing and PR-on wells with the exception of FPV showed full growth of yeast colonies, which were essentially the same as their respective PR-off control wells (data not shown). These results suggest that all FDA-approved PI drugs tested suppressed the inhibitory effect of HIV-1 PR on colony formation in fission yeast with the exception of FPV.

### 2.2. Effect of FDA-Approved Protease Inhibitor Drugs on HIV-1 Protease-Induced Growth Arrest

**Figure 3:**
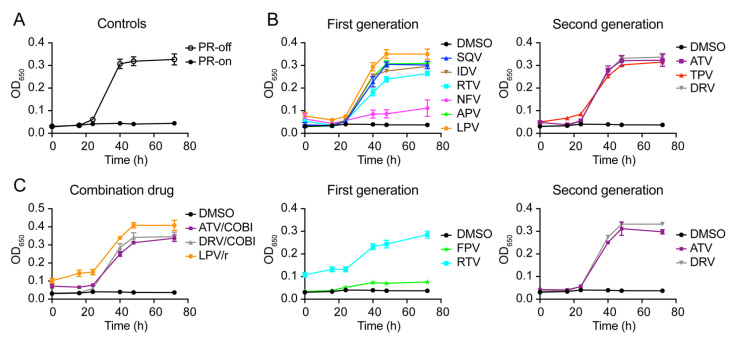
Suppression of HIV-1 PR-induced fission yeast cell growth arrest by FDA-approved PI drugs. (**A**) Assay controls: PR-off, no HIV-1 *PR* gene expression. PR-on, HIV-1 *PR* gene expression was induced hours (h) post-gene induction. (**B**) All PI drugs tested here were in pure chemical forms. (**C**) All PI drugs tested here were commercially available and formulated drug pills. To measure the effect of PI drugs on HIV-1 PR-induced cellular growth, HIV-1 *PR* gene was either induced (PR-on) or suppressed (PR-off) in RE294 cells in minimal PMG medium as described [25,32] in the presence or absence of drug treatment. A total of 150 μM of each drug was added. All liquid cell cultures were grown in 96-well microtiter plates in triplicates with the volume of 100 μL at 30 °C. Cell growth was measured overtime, as indicated by automated measurement of the OD_650_ using a spectrophotometer. Each datum is shown with an average and a standard error bar.

We next tested the potential effect of these PI drugs (150 μM) on HIV-1 PR-induced growth arrest as we reported previously [25,36]. Fission yeast cellular growth of the RE294 strain was measured over a time period of 72 h post-gene induction. As a control, growth of the RE294 cells was measured by an optical density (OD_650_)-based assay in the minimal and selective PMG liquid medium in the presence (PR-off) and absence (PR-on) of thiamine. As shown in Figure 3A, RE294 cells without HIV-1 PR protein production were grown normally with a typical sigmoid-shaped growth curve showing exponential growth during the log-phase. In contrast, little or no growth was observed in the PR-producing cells over the entire testing period.

Consistent with the fission yeast colony formation, DMSO had no effect on the cell growth, but all PI drugs in pure chemical forms restored cellular growth of the PR-producing RE294 cells (Figure 3B). Similarly, all PIs, with the exception of FPV, that were extracted from the commercially available and formulated drug pills also suppressed HIV-1 PR-induced growth arrest both in single-agent drugs and combination drugs (Figure 3C). Note that RTV, due to its reduced solubility, had higher background reading than others (Figure 3C). Together, these growth data in combination with the yeast colony formation data suggest that all FDA-approved PI drugs tested, except FPV, suppress HIV-1 PR-induced growth arrest in RE294 cells.

### 2.3. Effect of FDA-Approved Protease Inhibitor Drugs on Substrate Specific Proteolytic Cleavages of HIV-1 Protease in Fission Yeast

**Figure 4:**
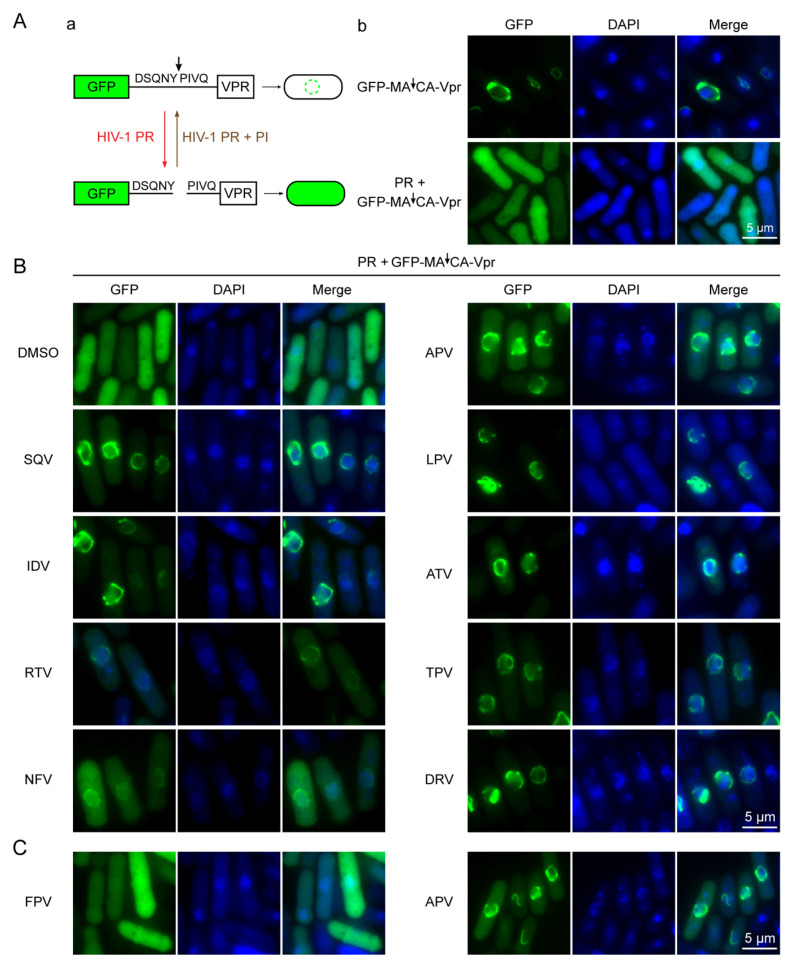
FDA-approved PI drugs prevent HIV-1 PR enzymatic cleavages of its nature HIV-1 substrate in fission yeast. (**A**) The schematic shows how the HIV-1 PR enzymatic assay is measured (**a**) with corresponding and expected results (**b**). (**B**) Results of each drug treatment. A total of 150 μM of each drug was used. Images were taken at 20 h post-gene induction. (**C**) Showing different results between the prodrug FPV (left) and drug APV (right). The measurement of HIV-1 PR-mediated proteolytic cleavage of a natural HIV-1 matrix-capsid (MA↓CA) substrate (DSQNY↓PIVQ) in fission yeast has been described previously [25,32]. Arrow indicates the PR cleavage site. GFP normally distribute uniformly throughout cells [39,40]. HIV-1 viral protein R (Vpr) localizes predominantly on the nuclear membrane and appears as a “ring-like” structure [39,40]. HIV-1 PR-mediated proteolytic cleavage of GFP-MA↓CA-Vpr will show uniform distribution of GFP; prevention of a PI drug on HIV-1 PR-mediated proteolytic cleavage will retain as a green “ring-like” structure, the same as without the production of HIV-1 PR [39,40].

The aim of this experiment was to test whether the suppressive effects of those FDA-approved PI drugs on the HIV-1 PR activities were mediated through the inhibition of its proteolytic enzymatic activity. The detailed method to measure the HIV-1 PR enzymatic activity in fission yeast cells has been described previously [25,32]. Briefly, a GFP- DSQNY↓PIVQ-Vpr fusion protein is used as the HIV-1 PR proteolytic target, in which DSQNY↓PIVQ is the natural HIV-1 MA↓CA substrate. GFP is used for fluorescent detection and it typically disperses throughout fission yeast cells [39,40]. HIV-1 Vpr is predominantly localized on the nuclear membrane in fission yeast [39,40] (Figure 4A). Consequently, the fusion protein production, without the HIV PR enzymatic cleavage, appears predominantly as a “ring-like” structure on the nuclear membrane because of the property of Vpr, i.e., the “Vpr pattern” [39,40]. In contrast, separation of GFP from Vpr by HIV-1 PR proteolytic cleavage of the substrate leads to the “GFP pattern” with uniform distribution throughout cells (Figure 4A) [39,40].

To examine the HIV-1 PR enzymatic activities, cells of a fission yeast strain G31, which is a derivative of RE294 cells containing the GFP-MA↓CA-Vpr fusion plasmid, were prepared as described [25,32] to induce HIV-1 *PR* gene expression and the production of the GFP-MA↓CA-Vpr fusion protein. Each PI drug was added to the PMG media right before the gene induction. Control and drug-treated G31 cells were collected 20 h post-gene induction. As shown in Figure 4B, adding DMSO to the gene-inducing G31 cells did not affect the PR enzymatic activity, as it showed a similar “GFP pattern”. Conversely, all PI-treated cells showed predominantly “ring-like” structures on the nuclear membranes, i.e., the “Vpr pattern”, suggesting they all inhibited the HIV-1 PR enzymatic activities (Figure 4B) [39,40]. These results suggest that the observed suppressive effects of those FDA-approved PI drugs on the HIV-1 PR activities were indeed mediated through the inhibition of its proteolytic enzymatic activities.

One of the possible reasons why FPV did not suppress the effect of HIV-1 PR on the colony formation (Figure 2) or the PR-induced cellular growth arrest (Figure 3) was probably because FPV is a phosphate ester prodrug of APV. After a patient takes FPV, FPV is converted to active APV by human alkaline phosphatases [41,42]. Here, we tested whether fission yeast has proper enzymes to process FPV to generate active PI activities. Figure 4C shows the comparison results of FPV- vs. APV-treated G31 cells. Consistent with the results of the other assays, APV suppressed the PR activities and showed the “ring-like Vpr pattern”, but FPV-treated cells showed no suppression with the “GFP pattern”, suggesting the HIV-1 PR enzymatic activities were still active and fission yeast does not have the proper enzymes to process the prodrug of FPV to generate APV.

### 2.4. Determination of Half Maximal Effective Concentration of FDA-Approved PI Drugs in Fission Yeast

**Figure 5:**
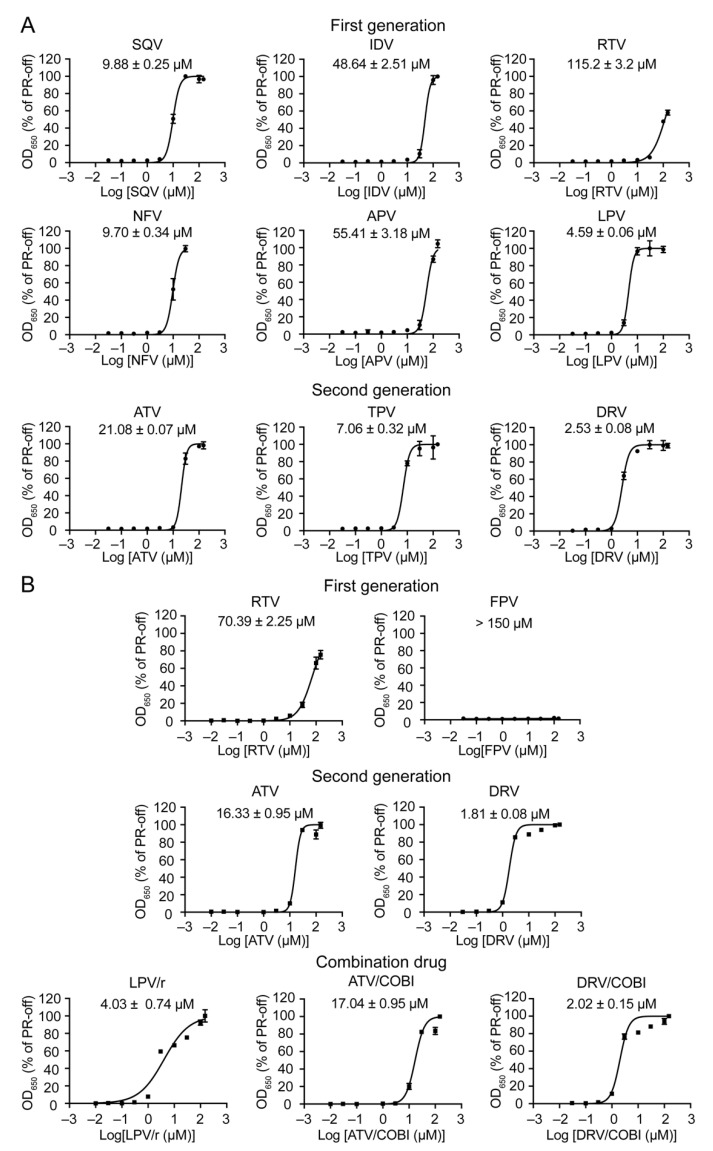
Determination of half maximal effective concentration of FDA-approved PI drugs in fission yeast. (**A**) Pure compound of PI drugs. (**B**) Formulated PI drug pills. HIV-1 *PR* gene expression was induced in RE294 cells, as described in Figure 2. The mean EC_50_ of each drug was measured in triplicate by a 9-dose (0.03, 0.1, 0.3, 1.0, 3.0, 10, 30, 100, and 150 μM) scheme. Cellular growth was measured at 72 h post-gene induction by optical density of 650 nm using a spectrophotometer. EC_50_ curves were generated by using Prism 8 software showing averages with standard error bars. The measurements were repeated with three different experiments.

The goal of this experiment was to determine the potency of each PI drug in fission yeast by the determination of EC_50_. EC_50_ refers here as the concentration of an FDA-approved PI drug to restore fission yeast growth of PR-producing cells halfway between the baseline and maximum growth of *PR*-suppressing cells, i.e., 50% of the *PR*-off cell control. To determine the EC_50_ of a PI drug, a dose-dependent curve was generated with a 9-dose (0.03, 0.1, 0.3 1.0, 3.0, 10, 30, 100, and 150 μM) drug treatment scheme. Note that due to the high cytotoxicity of NFV, only a lower and 7-dose testing scheme was used. Briefly, both HIV-1 *PR*-suppressing (*PR*-off) and *PR*-producing (*PR*-on) RE294 cells were prepared as described above. The PR-producing cells were added with a PI drug in the final concentration as indicated. Final cellular growth was measured at 72 h post-gene induction. The EC_50_ curve of each drug was generated by comparing the result of drug-treated PR-producing cells with that of the corresponding PR-off control cells to generate the relative % of cellular growth to the PR-off cells. The EC_50_s of each drug are shown in Figure 5 with the average values and standard derivations in micromolar concentration. These results show that each PI drug displays an about 2-log range of drug potencies in fission yeast, with DRV as the most potent (EC_50_ = 1.81 ± 0.08 μM) and RTV as the least strong (EC_50_ = 70.39 ± 2.25 μM). Note that, due to the high cytotoxicity of NFV, we had to remove the high concentration of 100 and 150 μM from data analyses. No EC_50_ value was assigned to FPV, as it had no inhibitory activity against the HIV-1 PR in fission yeast. When comparing the EC_50_ values between the pure compounds and the formulated drug forms, there were clear and statistically significant differences based on the pair-wise *t*-test analysis. The formulated drugs showed improved EC_50_ in comparison with the pure compounds. For example, the EC_50_ values decreased by 22.5% (ATV, *p* < 0.006), 28.5% (DRV, *p* < 0.001), and 38.9% (RTV, *p* < 0.001). However, including a booster regimen in the combination drugs did not improve the drug potencies of LPV/r, ATV/co, or DRV/co in fission yeast with their respective EC_50_ values of 4.59 ± 0.06 vs. 4.03 ± 0.74 for LPV/r, 16.33 ± 0.95 vs. 17.04 ± 0.95 for ATV/co, or 1.81 ± 0.08 vs. 2.02 ± 0.15 for DRV/co, respectively.

In summary, the PI drugs tested here showed a wide range of drug potencies in fission yeast with DRV as the most potent and RTV with the least strength. The EC_50_ values were significantly improved when the formulated drugs were used instead of the pure compounds. No significant differences were observed in the EC_50_ values when a booster of RTV or COBI was included in LPV/r, ATV/co, or DRV/co.

### 2.5. Effect of FDA-Approved Protease Inhibitor Drugs on a Multi-PI Drug Resistant HIV-1 Protease (_M11_PR) in Fission Yeast

We previously isolated several multi-PI resistant proteases from HIV-infected individuals [32]. Sanger sequencing by the clinical ViroSeq HIV-1 genotyping assay showed that one of them, _M11_PR, contains eleven drug resistant mutants (L10F, L33F, M46I, I54L, H69K, A71V, G73S, V77I, V82T, I84V, and L90M) in the HIV-1 *PR* encoding region. Phenotypic prediction suggests it might be resistant to all commercially available FDA-approved PI drugs with various degrees [32]. The goal here was to test the effect of FDA-approved PI drugs in our collection on this multi-PI drug resistant HIV-1 _M11_PR protease in fission yeast. A specially constructed fission yeast ZB011 strain was constructed for this study, which carries a single and integrated copy of the *_M11_PR* DNA fragment in the yeast chromosome.

**Figure 6:**
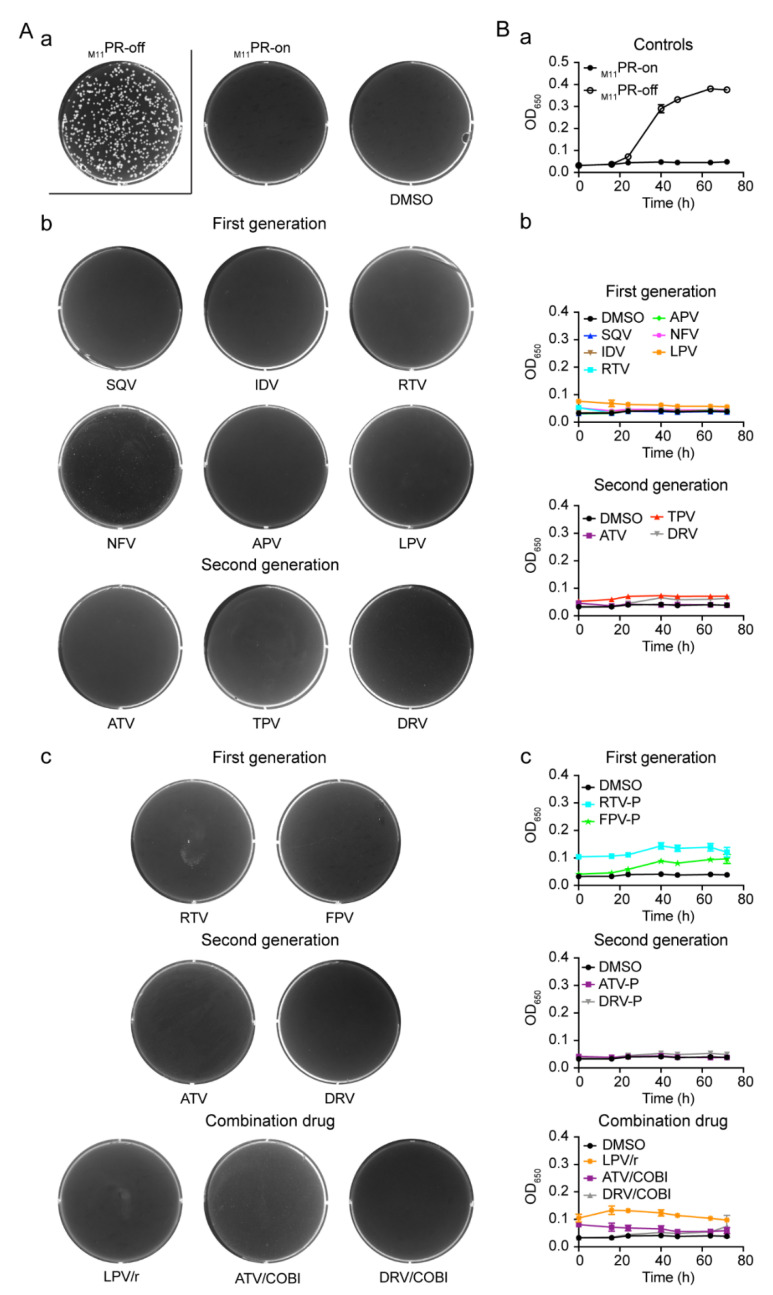
None of the FDA-approved PI drug suppress the activities of the multi-PI resistant HIV-1 _M11_PR mutant. (**A**) yeast colony formation of ZB011 cells. (**A-a**) Assay controls for yeast colony formation. (**A-b**) Results of cells treated with pure compounds. (**A-c**) Results of cells treated with formulated PI drugs. (**B**) Cellular growth of ZB011 over time. Pictures shown colony formation were taken at 4–5 days after incubation. Cell growth was measured over time as indicated by automated measurement of OD_650_ using a spectrophotometer. Each datum is shown with an average and standard error bar.

The same fission yeast colony formation assay and the growth curve analysis were used to test each of the PI drugs against the ZB011 strain. All the experimental conditions were also the same as described above for the tests for the wild-type PR. As shown in Figure 6A-a, both the positive and negative controls worked properly, and DMSO had no effect on the _M11_PR-producing cells. Unlike what was shown in Figure 2 for the colony formation, however, none of the PI-treated ZB011 wells showed any visible colony formation with either the pure compounds (Figure 6A-b) or the formulated drug pills (Figure 6A-c). Similarly, none of the PIs were able to suppress _M11_PR-induced cellular growth arrest over time (Figure 6B-b,c), although the _M11_PR without drug treatment was able to completely block the cellular growth of the ZB011 cells (Figure 6B-a). These results suggest that, consistent with the early prediction by the ViroSeq HIV-1 genotyping test, the _M11_PR is indeed resistant to all of the FDA-approved PI drugs tested in fission yeast.

## 3. Discussion

In this study, we tested all thirteen FDA-approved HIV-1 PI drugs (Table 1; Figure 1) in two fission yeast cell-based systems that we previously developed and designed to test the wild-type HIV-1 PR [25,36] and the multi-PI drug resistant _M11_PR [32]. Our results showed that all FDA-approved single-agent PI drugs, except for FPV, were able to suppress the wild-type PR activities in the forms of yeast colony formation (Figure 2), cellular growth arrest (Figure 3), and proteolytic enzyme cleavage of a natural HIV-1 substrate MA↓CA (DSQNY↓PIVQ) (Figure 4). Moreover, the EC_50_ range was about 2-logs in both fission yeast and human cells with DRV as the strongest and RTV as the weakest (Figure 5). The EC_50_ of the combination drug showed essentially the same potency as its respective single-agent drug. In contrast to what was observed in the wild-type HIV-1 PR, none of the FDA-approved drugs were able to suppress any of the presumably multi-PI drug resistant _M11_PR activities (Figure 6).

The fission yeast cell-based systems used in this study have been described previously [25,32]. Several FDA-approved single-agent PI drugs have been used in those initial proof-of-concept studies. The goal of this study was to further demonstrate the utility of the established fission yeast cell-based systems in testing future HIV-1 protease inhibitors by using different types of FDA-approved PI drugs as examples. In addition, we wanted to quantify the drug potencies in fission yeast cells by the measurement of EC_50_ and further compared them in pure compounds, formulating single-agent drug and combination drugs mixed with a booster of human cells.

Our results showed that all the FDA-approved PI drugs tested worked as expected in fission yeast with the exception of FPV, which did not show any inhibitory effect against the wild-type PR. FPV is used in HIV treatment as a phosphate ester prodrug of APV [41,43]. In the human body, FPV must be first converted to APV by alkaline phosphatases to form the active agent [41,43]. One of the reasons why we included FPV here was to test whether fission yeast has a similar enzyme to process FPV. Our results showed that FPV did not exhibit any PI activity in fission yeast, suggesting that fission yeast does not have the proper enzymes to convert FPV to APV as the human body does. It would be interesting to test in the future whether we could activate the PI activity of FPV by adding the human alkaline phosphatase to fission yeast cells.

Interestingly, the EC_50_ values of single-agent drugs were improved more significantly in the formulated drug pills than in the pure compounds. Among the three drugs tested, the EC_50_ improved significantly by 22.5% (ATV, *p* < 0.006), 28.5% (DRV, *p* < 0.001), and 38.9% (RTV, *p* < 0.001), respectively (Figure 5). Generally, there are many different inactive ingredients or drug carriers in a formulated drug pill to enhance the drug load solid dissolution rate. Thus, it would be difficult to sort out which one of those components contribute to the improved EC_50._ However, in the case of DRV or RTV, gelatin 50PS and bovine serum albumin (BSA) have been shown to improve their drug solubilities [44]. Therefore, it would be of interest to test whether gelatin 50PS or BSA is the potential contributing factor to improve the drug uptakes by fission yeast.

However, including a booster regimen in the combination drugs did not improve the EC_50_ of LPV/r, ATV/co, or DRV/co. Note that the purpose of including a booster regimen in the combination drug in treating HIV infection is to enhance the drug efficacy. For example, in the LPV/r combination, RTV is used as a pharmacokinetic enhancer because the low dose of RTV inhibits cytochrome CYP3A4-mediated LPV metabolism and the P-glycoprotein efflux pump, thereby providing effective LPV plasma exposure [45]. Similarly, COBI is also used to inhibit human CYP3A4 enzymes in the combination of ATV/co or DRV/co [46,47]. By including COBI with ATV or DRV, inhibition of human CYP3A enzymes could result in a relatively higher drug dose in the body than without COBI, thus improving drug efficacy. However, COBI by itself does not have any antiviral activity or ability to interfere with HIV PR activity. Since fission yeast has two endogenous CYP enzymes (CYP51 and CYP61) [48], our goal here was to examine whether those fission yeast CYPs have intrinsic drug metabolizing enzymes to boost the drug efficacy with a pK enhancer. However, neither RTV nor COBI improved the drug potency in the combination drugs in fission yeast cells, as no differences in the EC_50_ values were observed between a single-agent drug and its respective combination drug, e.g., 4.59 ± 0.06 vs. 4.03 ± 0.74 for LPV/r, 16.33 ± 0.95 vs. 17.04 ± 0.95 for ATV/co, or 1.81 ± 0.08 vs. 2.02 ± 0.15 for DRV/co, respectively (Figure 5). These data suggest that fission yeast does not have the proper CYP drug metabolizing enzymes to boost the drug efficacy. Although yeasts do not produce human equivalent CYP3A4 homologous enzymes, all human drug-metabolizing P450 enzymes including CYP3A4 are functional in fission yeast [49] and budding yeast [50]. In fact, both yeasts have established systems to stably expresses human CYP3A4 for the studies of drug metabolism [49,51,52]. Therefore, it would be of interest to test in the future whether we could adapt those humanized fission yeast systems with stably expressed HIV-1 PR and human CYP3A4 for the studies of not only PI activity but also drug efficacy with a pK enhancer. In addition, it would be interesting to test the combinational effect of different PI drugs and to see which PI combination could potentially show synergistic effect and possibly circumvent multi-PI drug resistance.

The single-agent PI drug showed a wide range (about 2-logs) of different EC_50_ in fission yeast (Figure 5). Interestingly, a similar 2-log range of the EC_50_ was also seen in human cells (Table 2). Furthermore, DRV showed the most potent EC_50_ and RTV showed the least potent EC_50_ in both human and fission yeast cells (Figure 5; Table 2). These data suggest that all PI drugs behaved similarly in fission yeast and human cells in suppressing the HIV-1 PR activities. However, it required a much higher drug concentration to achieve the same level of HIV protease inhibition in fission yeast than in human cells. Note that the EC_50_ measured in fission yeast does not evaluate the antiviral activity of the PI drugs against HIV, but rather only their inhibitory effect on the HIV PR activity. In contrast, EC_50_ measured in human cells is based on antiviral activity against the entire virus. Thus, it is not appropriate to compare the absolute EC_50_ values but rather to provide an estimation of the differences between the two cell-based systems. Alternatively, all the PI drugs tested here have also been measured in other studies by in vitro HIV-1 PR enzymatic assays [8,53]. Similar to the EC_50_ values measured in human cells, EC_50_ values measured based on in vitro HIV-1 PR enzymatic assays are also in the range of nM [8,53]. Overall, the difference of EC_50_ values between fission yeast and human cells is about 2–3 logs. For example, the EC_50_ of DRV in human cells is approximately 1–10 nM [54], but it took 1.8–2.5 μM to achieve the same EC_50_ in fission yeast cells. These differences in EC_50_ are likely attributed to the fact that fission yeast has a thick cell wall, which could result in less efficient drug uptake than human cells. If this were true, removing yeast cell walls by generating spheroplasts could potentially improve the drug uptake. Early studies showed that cryopreserved spheroplasts remain competent with high viability over many months [55,56]. Tests are currently underway to test this hypothesis. Alternatively, fission yeast could have a more efficient efflux pump system for smaller molecule compounds, such as the PI drugs, than human cells. Fission yeast has two types of drug efflux pumps, which include 11 ATP-binding cassettes (ABCs) and 49 major facilitator superfamily (MFS) transporters [57,58]. Combinations of different ABC and MFS mutant strains have been created that are defective in uptaking chemical compounds [58,59,60]. It would be interesting to test which one of those ABC/MFS defective strains could increase the uptake of the PI drugs. The third possibility is that the level of HIV-1 PR protein produced in fission yeast is higher than that in infected human cells, because the *nmt*1 promoter we used in this study is a strong promoter [37]. Consequently, higher PI drug concentration might be needed to suppress the HIV-1 PR activities. The level of *nmt*1 transcriptional activities can be titrated down by increasing the amount of thiamine used (our unpublished data). Alternatively, different strengths of *nmt*1 promoters are also available [37,38]. It would be interesting to test in the future whether this is the case. It is worthwhile mentioning that even though it needs higher drug concentration to achieve the same EC_50_ in fission yeast than in human cells, it could potentially be used as a way to eliminate background in high-throughput drug screenings and to select for those protease inhibitors that are highly potent, thus providing better lead candidates for the development of new drugs.

The main function of HIV-1 PR is to cleave HIV-1 Gag/Pol polyproteins, yet Gag/Pol polyproteins are not present in the fission yeast system. One might ask what is the molecular action of HIV-1 PR-induced growth inhibition and cell death? Are there any peptide sequences similar to the PR HIV-1 cleavage sites in the fission yeast genes? What is the target(s) of HIV-1 PR in fission yeast? Currently, no known peptide sequences similar to the PR HIV-1 cleavage sites in the fission yeast genes are found, nor was there any known and specific cellular target(s) of HIV-1 PR in fission yeast. The main challenge of finding specific peptide substrate(s) or cellular protein target(s) of HIV-1 PR in fission yeast or human cells is that HIV-1 substrates themselves share little sequence identity or consensus binding motif. Rather, the overall geometric structure of the PR-substrate complex is more relevant [4,69]. BlastP searches using the HIV-1 MA substrate (DSQNYPIVQ) as a query peptide, which was used in this study, against the fission yeast databases yielded no meaningful results. However, in one of our earlier studies and through genome-wide searches of HIV-1 PR cellular suppressive proteins in fission yeast, we identified a fission yeast serine/threonine kinase Hhp2, a human homolog of CK1α [25]. When it was overproduced, it not only suppresses HIV-1 PR-induced cell death in fission yeast, but also prevents PR-induced apoptosis and cleavage of caspase-3 and caspase-8 in human cells [25]. These findings, along with the fact that HIV-1 PR does cleave specifically the HIV-1 substrates in fission yeast cells, support that the described fission yeast cell-based systems are suitable systems for the study of HIV-1 PR [4,25,32,36].

Overall, as we have demonstrated previously [4,25,36,70,71], the results of this study once again support the idea that fission yeast could be used as a reliable system for the discovery and testing of HIV-1 protease inhibitors. There are a number of advantages of using a fission yeast cell-based system for future drug discovery and testing. First, fission yeast is a single eukaryotic organism that shares many highly conserved basic cell activities such as cell proliferation, cell cycle regulation, and regulation of cell death [71,72,73]. It is present mostly in its haploid state, which makes it easier to study the genetic effect of interest by avoiding high genetic complexity such as the dominance or recession of a genetic trait in human cells. It is easy to culture fission yeast cells in the laboratory with a short cell-doubling time, so it is fast, economic, and amendable for large-scale drug screening and testing. Most importantly, in contrast to structure-based drug designs, such a fission yeast cell-based system has all of the advantages of a cell-based assay, i.e., (1) cytotoxic compounds can be automated removed from the drug screening, (2) it is easy to maintain and cost-effective, and (3) it could be used to eliminate background and select only for only those highly potent drug candidates. In addition, such a fission yeast cell-based system has no presumption of what type of PIs will be discovered. Thus, it has the potential to uncover novel PIs that not only inhibit the enzymatic site, i.e., the competitive inhibitors, but also target other regions of the PR, i.e., the allosteric inhibitors. The described fission yeast cell-based systems also have their shortcomings. Besides the differences of the intracellular uptakes and drug efflux systems between human and yeast cells, they require higher drug concentration to achieve the same EC_50_ in fission yeast than in human cells. This reduced sensitivity could potentially miss promising lead PI compounds during large-scale drug screenings. Nevertheless, as the emergence of viral multi-PI drug resistance arises, the described fission yeast _M11_PR system will be useful in future screening and testing of potential PIs against multi-drug resistant PRs. Note that, so far, no PI has yet been discovered to suppress the _M11_PR activities and other naturally occurring drug resistant PRs [19,20,21,25,74,75,76], which provides a strong argument that there is a need to discover new and more potent antiretroviral PI drugs than what we have today in order to continue combating future and emerging HIV multidrug resistance.

## 4. Materials and Methods

### 4.1. Fission Yeast Strains and Inducible Gene Expression

A commonly used wild-type fission yeast (*Schizosaccharomyces pombe*) strain SP223 (*h-, ade6-216, leu1-32, ura4-294)* was used to generate all the other strains used in this study [77]. RE294 (*h-, ade6-216 leu1-32 ura4-294, nmt1*-pr(NL4-3)-kanMX) is a derivative of SP223 strain that contains a single integrated copy of the HIV-1 *PR* gene at the *nmt1* gene locus [25,36]. ZB011 (*aka* _M11_PR or ZB011-PRM11) is another derivative of SP223 strain (*h-, ade6-216 leu1-32 ura4-294*::pCloneBle1-Δura4-proteaseM11) that contains a single integrated copy of a drug resistant HIV-1 _M11_*PR* mutant gene at the *ura4* gene locus. This mutant _M11_PR was originally isolated from a HIV-infected individual at the University of Maryland Medical Center [32]. Sanger sequencing by the clinical ViroSeq assay shows that it contains eleven drug resistant mutants (L10F, L33F, M46I, I54L, H69K, A71V, G73S, V77I, V82T, I84V, and L90M), which were predicated to be resistant to all commercially available PI drugs approved by the FDA [32]. G31 is a derivative of RE294 that carries a pYZ3N-GFP-MA↓CA-Vpr plasmid expressing a GFP-MA↓CA-Vpr fusion protein [25].

All fission yeast cells were grown in minimal Pombe Glutamate Medium (PMG) supplemented with 225 μg/mL of adenine, uracil, leucine, thiamine, or G418 based on the auxotrophic markers for yeast cell growth and plasmid selections as described previously [25,32,36]. RE294 cells were grown in PMG supplemented with adenine, uracil, leucine, and geneticin (G418). ZB011 was grown in PMG supplemented with adenine, uracil, and leucine under the same condition, except Zeocin was used as the selection marker instead of G418 [25,78]. G31 was supplemented with adenine, uracil, and G418. HIV-1 *PR* gene expression is under the control of an inducible no message in thiamine (*nmt1)* promoter [37]. Specifically, the starting fission yeast cell culture in the presence of 20 μM thiamine (to prevent *nmt1*-mediated gene expression) was first inoculated and refreshed by overnight growth at 30 °C to obtain active growing cells. Cells were washed three times with distilled water and diluted cells to a final concentration of 2 × 10^4^ cells/mL in PMG with 20 μM thiamine (PR-off) or without thiamine (PR-on). Full gene expression can be achieved in about 16 h post-gene induction [37,79].

### 4.2. FDA-Approved HIV Protease Inhibitor Drugs

A list of FDA-approved HIV-1 PI drugs that are used in this study are summarized in Table 1. Chemical structures of these PI drugs are listed in Figure 1. These PIs were tested in two different forms, i.e., pure compounds or formulated drug pills. All these PI drugs were rationally designed to mimic the transition state of the polyprotein substrates of HIV-1 protease [34,53]. Both generations of PI drugs as described previously [8] are tested here, i.e., the first-generation PI drugs are competitive peptidomimetic inhibitors and the second-generation PI drugs are non-peptidyl P2-ligand-based inhibitors that inhibit PR dimerization with a higher barrier to resistance [8]. A combination drug is a formulated and fixed-dose drug containing more than one PI inhibitors (e.g., LPV/r) or a PI drug with a PI booster (e.g., ATV/co or DRV/co). Note that FPV is the phosphate ester prodrug of APV. After taking the drug, FPV is hydrolyzed to APV in the gut epithelium by cellular alkaline phosphatases [42], making FPV a slow-releasing APV drug in order to prolong the drug efficacy [41]. All the PI drugs used in this study were dissolved in DMSO.

### 4.3. Fission Yeast Colony Formation Assay

A modified fission yeast colony-formation assay was used to investigate the effect of the PR gene expression on fission yeast cell proliferation from previously described protocol [25,32]. Briefly, instead of using regular agar plate, a 6-well culture plate (REF: 3516, Corning Inc., Kennebunk, ME, USA) was used in order to reduce the amount of the drugs used. Specifically, minimal and thiamine-free PMG liquid media containing 2% agar with proper supplementation of auxotrophic mutants and G418 or Zeocin antibiotic were used to make the mini-agar wells. A total of 150 μM of each drug was mixed with the agar-containing PMG media at 60 °C before adding to each well. HIV-1 *PR*-inducing RE294 or ZB011 cells were prepared the same way as described in Section 4.1 [25,32]. A total number of 1 × 10^3^ HIV-1 *PR*-inducing cells with the volume of 50 μL were added to solidified agar in each well. A plastic rod was used to spread cells evenly on the surface of the agar wells. The inhibitory effect of each FDA PI drug on yeast colony formation was evaluated 4–5 days after plating by comparing the growth of the colonies of the drug-treated wells to that of the mock DMSO controls.

### 4.4. Fission Yeast Growth Assay

The assay used to measure cellular growth in fission yeast cells has been described previously [25,80]. Briefly, fission yeast cells were grown in the minimal PMG media. RE294 or ZB011 cells carrying a single and integrated HIV-1 *PR* or _M11_*PR* encoding sequence in the chromosome that is under the control of a *nmt1* promoter were maintained selectively in appropriately supplemented media with 20 μM thiamine to silence gene expression. For gene induction, cells were first grown to mid-log growth phase in the presence of 20 μM thiamine. Cells were then washed three times with distilled water and diluted to a final concentration of approximately 2 × 10^4^ cells/mL in PMG media supplemented with thiamine (PR-off) or without thiamine (PR-on). A total of 100 μL/well of cell culture was dispensed to a 96-well microtiter plate and incubated at 30 °C. Cell growth was measured at each time point as indicated by automated measurement of the optical density (OD_650_) using a spectrophotometer.

### 4.5. Measurement of HIV PR Enzymatic Activity in Fission Yeast

A “green fluorescent protein (GFP) re-localization assay” that was developed in our laboratory [25,32] was used to measure the specific proteolytic cleavage of an HIV-1 natural substrate MA↓CA (DSQNY↓PIVQ) by HIV-1 PR. Specifically, to test the effect of a PI drug on HIV-1 PR enzymatic activity, G31 cells were prepared as described above to induce HIV-1 *PR* gene expression and the production of the GFP-MA↓CA-Vpr fusion protein in fission yeast cells. Each PI drug was added at the concentration of 150 μM to the PMG media. Control and drug treated G31 cells were collected 20 h post gene induction. As result, the production of the GFP-MA↓CA-Vpr fusion protein in fission yeast cells appears predominantly as a “ring-like” structure on the nuclear membrane because of the property of Vpr [39,40]. In the presence of HIV-1 PR, the proteolytic cleavage of the MA↓CA will separate GFP from Vpr, leading to uniform distribution of GFP throughout cells [39,40]. However, if a PI drug inhibits the HIV-1 PR enzymatic activity in fission yeast, we would expect restoration of a “ring-like” structure of the GFP-MA↓CA-Vpr fusion protein on the nuclear membrane. DAPI (4′,6′-diamidino-2-phenylindole) (Vector Laboratory Inc) was used to stain nuclei as a reference. A Leica fluorescence microscope DMR4500B equipped with a high-performance CCD camera (Hamamatsu) and Open-Lab software (Improvision, Inc., Lexington, MA) was used for fluorescence imaging analyses. Fixed fission yeast sample with 4% (w/v) formaldehyde was first stained with 1 μg/mL DAPI and then placed onto a regular glass slide and covered with a cover slip. A Leica L5 filter with excitation of 480/40 and emission of 527/30 was used to observe green fluorescence, and a Leica A8 filter with an excitation of 360 nm (range, 340 to 380 nm) and emission of 470 nm (range, 450 to 490 nm) was used to locate nuclei.

### 4.6. Measurement of Half Maximal Effective Concentration of FDA-Approved PI Drugs in Fission Yeast

Half maximal effective concentration (EC_50_) refers here to the concentration of an FDA-approved PI drug that restores fission yeast cellular growth halfway between the baseline and maximum growth of HIV-1 *PR*-suppressing cells, i.e., 50% of the *PR*-off cell control. To determine the EC_50_ of a PI drug, a dose-dependent curve was generated with a 9-dose (0.01, 0.1, 0.3, 1.0, 3.0, 10, 30, 100, and 150 μM) drug treatment scheme. Briefly, both HIV-1 *PR*-suppressing (*PR*-off) or *PR*-producing (*PR*-on) RE294 cells were prepared as described above as control and for testing. The PR-producing cells were added with a PI drug in the final concentration as shown. Final cellular growth was measured at 72 h post-gene induction by optical density of 650 nm using a spectrophotometer. The EC_50_ curve of each drug was generated by comparing the result of drug-treated PR-producing cells with that of PR-off control cells to generate the relative % of cellular growth to the PR-off cells.

### 4.7. Statistical Analysis

Pair-wise *t*-test analysis was used in this study to test potential statistically significant differences of EC_50_ between the pure compounds and the formulated drugs by using the software Prism 8 (GraphPad, San Diego, CA, USA). Statistical significance was accepted at the 95% confidence level (*p* < 0.05).

## Figures and Tables

**Table 1:** FDA-approved HIV-1 protease inhibitor drug used in this study.

Drug Name	Abbreviation	Brand Name	Molecular Weight (g/mol) **	Drug Manufacturer	Pure Compound Source ^‡^ (ARP#)
First-generation single-agent drug					
Saquinavir	SQV	Invirase	670.84	F. Hoffmann-La Roche	4658
Indinavir	IDV	Crixivan	613.79	Merck	8145
Ritonavir	RTV	Norvir	720.94	Abbott Labs	4622
Nelfinavir	NFV	Viracept	567.78	Pfizer	4621
Amprenavir	APV	Agenerase	505.63	Vertex Pharmaceuticals	8148
Fosamprenavir *	FPV	Lexiva	585.61	GlaxoSmithKine	na
Lopinavir	LPV	Kaletra	628.81	Abbott Labs	9481
Second-generation single-agent drug					
Atazanavir	ATV	Reyataz	704.86	Bristol-Myers Squibb	10,003
Tipranavir	TPV	Aptivus	602.66	Boehringer Ingelheim	11,285
Darunavir	DRV	Prezista	547.66	Tibotec	11,447
Fixed dose combination drug					
Lopinavir /Ritonavir	LPV/r	Kaletra	628.81 /720.94	Abbott Labs	na
Atazanavir/ Cobicistat	ATV/co	Evotaz	704.86	Bristol-Myers Squibb	na
Darunavir/ Cobicistat	DRV/co	Prezcobix	547.66	Tibotec	na

**Note:** These PI drugs are listed in the order of years of FDA approval. *, FPV is prodrug of APV; **, from go.drugbank.com (accessed on 02/03/2021); **^‡^**, all pure compounds were from the NIH HIV Reagent Program (ARP), Division of AIDS, NIAID, NIH; na, not available; The first-generation PI drugs are defined as those (1) competitive peptidomimetic inhibitors, (2) peptidyl inhibitor based on hydroxyethylene and hydroxyethylamine isosteres, and (3) the central hydroxyl group mimics the transition state of the hydrolysis step by binding with the catalytic aspartic acid residues [8]. The second-generation PI drugs are referring those (1) non-peptidyl inhibitor, (2) dimerization inhibitors, (3) G2 ligand, and/or (4) higher barrier to resistance [8]. A combination drug is a formulated drug containing more than one PI inhibitor (e.g., LPV/r) or a PI drug with a PI booster (e.g., ATV/co or DRV/co).

**Table 2:** Half maximal effective concentration of HIV PI drugs in human and fission yeast cells.

HIV-1 Protease Inhibitor Drug	EC_50_ in Human Cell (nM) ^1^	EC_50_ in Fission Yeast /Pure Compound (μM) ^2^	EC_50_ in Fission Yeast /Drug Formulation (μM) ^3^	Reference
First-generation single-agent drug				
Saquinavir (SQV)	5–25	9.88 ± 0.25	nd	[61]
Indinavir (IDV)	5–30	48.64 ± 2.51	nd	[62]
Ritonavir (RTV)	40–100	115.2 ± 3.2	70.39 ± 2.25	[53,63]
Nelfinavir (NFV)	3–40	9.7 ± 0.34	nd	[53,63]
Amprenavir (APV)	15–60	55.41 ± 3.18	nd	[18]
Fosamprenavir (FPV) *	cnd	na	cnd	[18]
Lopinavir (LPV)	4–11	4.59 ± 0.06	nd	[64]
Second-generation single-agent drug				
Atazanavir (ATV)	2–5	21.08 ± 0.07	16.33 ± 0.95	[65]
Tipranavir (TPV)	30–70	7.86 ± 0.32	nd	[66]
Darunavir (DRV)	1–10	2.53 ± 0.08	1.81 ± 0.08	[54]
Fixed dose combination drug				
Lopinavir/Ritonavir (LPV/r)	na	na	4.03 ± 0.74	[67]
Atazanavir/ Cobicistat (ATV/co)	na	na	17.04 ± 0.95	[68]
Darunavir/Cobicistat (DRV/co)	1–2	na	2.02 ± 0.15 *	[68]

**Note:** *, FPV is prodrug of APV; ^1^, from [53,63]; ^2^, pure compounds; ^3^, compounds in drug pill forms; EC_50_, mean 50% effective concentration; na, not available; nd, not determined; cnd, cannot be determined. LPV/r, LPV, and RTV combination. Cobicistat, an inhibitor of human CYP3A proteins (under the brand name Tybost). It is used to boost the PI effect.

## Data Availability

Not applicable.

## References

[1] Jackson S.S., Sumner L.E., Finnegan M.A., Billings E.A., Huffman D.L., Rush M.A. (2020). A 35-year review of pre-clinical HIV therapeutics research reported by NIH ChemDB: Influences of target discoveries, drug approvals and research funding. J. AIDS Clin. Res..

[2] Markham A. (2020). Fostemsavir: First approval. Drugs.

[3] Bhatti A.B., Usman M., Kandi V. (2016). Current scenario of HIV/AIDS, treatment options, and major challenges with compliance to antiretroviral therapy. Cureus.

[4] Yang H., Nkeze J., Zhao R.Y. (2012). Effects of HIV-1 protease on cellular functions and their potential applications in antiretroviral therapy. Cell Biosci..

[5] Calugi C., Guarna A., Trabocchi A. (2013). Heterocyclic HIV-protease inhibitors. Curr. Med. Chem..

[6] Pang X., Liu Z., Zhai G. (2014). Advances in non-peptidomimetic HIV protease inhibitors. Curr. Med. Chem..

[7] Mallolas J. (2017). Darunavir stands up as preferred HIV protease inhibitor. AIDS Rev..

[8] Ghosh A.K., Osswald H.L., Prato G. (2016). Recent progress in the development of HIV-1 protease inhibitors for the treatment of HIV/AIDS. J. Med. Chem..

[9] Wlodawer A., Miller M., Jaskolski M., Sathyanarayana B.K., Baldwin E., Weber I.T., Selk L.M., Clawson L., Schneider J., Kent S.B. (1989). Conserved folding in retroviral proteases: Crystal structure of a synthetic HIV-1 protease. Science.

[10] Persaud D., Siberry G.K., Ahonkhai A., Kajdas J., Monie D., Hutton N., Watson D.C., Quinn T.C., Ray S.C., Siliciano R.F. (2004). Continued production of drug-sensitive human immunodeficiency virus type 1 in children on combination antiretroviral therapy who have undetectable viral loads. J. Virol..

[11] Combescure C., Vallier N., Ledergerber B., Cavassini M., Furrer H., Rauch A., Battegay M., Bernasconi E., Vernazza P., Hirschel B. (2009). How reliable is an undetectable viral load?. HIV Med..

[12] Ghosh A.K., Thompson W.J., McKee S.P., Duong T.T., Lyle T.A., Chen J.C., Darke P.L., Zugay J.A., Emini E.A., Schleif W.A. (1993). 3-Tetrahydrofuran and pyran urethanes as high-affinity P2-ligands for HIV-1 protease inhibitors. J. Med. Chem..

[13] Brunton L.L., Lazo J.S., Parker K.L. (2006). Goodman and Gilman’s The Pharmacological Basis of Therapeutics.

[14] Laco G.S., Schalk-Hihi C., Lubkowski J., Morris G., Zdanov A., Olson A., Elder J.H., Wlodawer A., Gustchina A. (1997). Crystal structures of the inactive D30N mutant of feline immunodeficiency virus protease complexed with a substrate and an inhibitor. Biochemistry.

[15] Ghosh A.K., Anderson D.D., Weber I.T., Mitsuya H. (2012). Enhancing protein backbone binding—A fruitful concept for combating drug-resistant HIV. Angew. Chem. Int. Ed..

[16] Deeks E.D. (2014). Darunavir: A review of its use in the management of HIV-1 infection. Drugs.

[17] Zeldin R.K., Petruschke R.A. (2004). Pharmacological and therapeutic properties of ritonavir-boosted protease inhibitor therapy in HIV-infected patients. J. Antimicrob. Chemother..

[18] Barbour A.M., Gibiansky L., Wire M.B. (2014). Population pharmacokinetic modeling and simulation of amprenavir following fosamprenavir/ritonavir administration for dose optimization in HIV infected pediatric patients. J. Clin. Pharmacol..

[19] Palmer S., Shafer R.W., Merigan T.C. (1999). Highly drug-resistant HIV-1 clinical isolates are cross-resistant to many antiretroviral compounds in current clinical development. AIDS.

[20] Logsdon B.C., Vickrey J.F., Martin P., Proteasa G., Koepke J.I., Terlecky S.R., Wawrzak Z., Winters M.A., Merigan T.C., Kovari L.C. (2004). Crystal structures of a multidrug-resistant human immunodeficiency virus type 1 protease reveal an expanded active-site cavity. J. Virol..

[21] Rhee S.Y., Taylor J., Fessel W.J., Kaufman D., Towner W., Troia P., Ruane P., Hellinger J., Shirvani V., Zolopa A. (2010). HIV-1 protease mutations and protease inhibitor cross-resistance. Antimicrob. Agents Chemother..

[22] Ssemwanga D., Lihana R.W., Ugoji C., Abimiku A., Nkengasong J., Dakum P., Ndembi N. (2015). Update on HIV-1 acquired and transmitted drug resistance in Africa. AIDS Rev..

[23] Tang M.W., Shafer R.W. (2012). HIV-1 antiretroviral resistance: Scientific principles and clinical applications. Drugs.

[24] Kozyryev I., Zhang J. (2015). Bayesian analysis of complex interacting mutations in HIV drug resistance and cross-resistance. Adv. Exp. Med. Biol..

[25] Benko Z., Elder R.T., Li G., Liang D., Zhao R.Y. (2016). HIV-1 Protease in the Fission Yeast *Schizosaccharomyces pombe*. PLoS ONE.

[26] Sanchez P.R., Holguin A. (2014). Drug resistance in the HIV-1-infected paediatric population worldwide: A systematic review. J. Antimicrob. Chemother..

[27] Cote H.C., Brumme Z.L., Harrigan P.R. (2001). Human immunodeficiency virus type 1 protease cleavage site mutations associated with protease inhibitor cross-resistance selected by indinavir, ritonavir, and/or saquinavir. J. Virol..

[28] Wu T.D., Schiffer C.A., Gonzales M.J., Taylor J., Kantor R., Chou S., Israelski D., Zolopa A.R., Fessel W.J., Shafer R.W. (2003). Mutation patterns and structural correlates in human immunodeficiency virus type 1 protease following different protease inhibitor treatments. J. Virol..

[29] Kantor R., Fessel W.J., Zolopa A.R., Israelski D., Shulman N., Montoya J.G., Harbour M., Schapiro J.M., Shafer R.W. (2002). Evolution of primary protease inhibitor resistance mutations during protease inhibitor salvage therapy. Antimicrob. Agents Chemother..

[30] Voshavar C. (2019). Protease inhibitors for the treatment of HIV/AIDS: Recent advances and future challenges. Curr. Top. Med. Chem..

[31] Vickrey J.F., Logsdon B.C., Proteasa G., Palmer S., Winters M.A., Merigan T.C., Kovari L.C. (2003). HIV-1 protease variants from 100-fold drug resistant clinical isolates: Expression, purification, and crystallization. Protein Expr. Purif..

[32] Benko Z., Liang D., Li G., Elder R.T., Sarkar A., Takayama J., Ghosh A.K., Zhao R.Y. (2017). A fission yeast cell-based system for multidrug resistant HIV-1 proteases. Cell Biosci..

[33] Koh Y., Aoki M., Danish M.L., Aoki-Ogata H., Amano M., Das D., Shafer R.W., Ghosh A.K., Mitsuya H. (2011). Loss of protease dimerization inhibition activity of darunavir is associated with the acquisition of resistance to darunavir by HIV-1. J. Virol..

[34] Aoki M., Das D., Hayashi H., Aoki-Ogata H., Takamatsu Y., Ghosh A.K., Mitsuya H. (2018). Mechanism of darunavir (DRV)’s high genetic barrier to HIV-1 resistance: A key V32I substitution in protease rarely occurs, but once it occurs, it predisposes HIV-1 to develop DRV resistance. mBio.

[35] Kneller D.W., Agniswamy J., Harrison R.W., Weber I.T. (2020). Highly drug-resistant HIV-1 protease reveals decreased intra-subunit interactions due to clusters of mutations. FEBS J..

[36] Benko Z., Zhang J., Zhao R.Y. (2019). Development of A fission yeast cell-based platform for high throughput screening of HIV-1 protease inhibitors. Curr. HIV Res..

[37] Maundrell K. (1993). Thiamine-repressible expression vectors pREP and pRIP for fission yeast. Gene.

[38] Zhao Y., Elder R.T., Chen M., Cao J. (1998). Fission yeast expression vectors adapted for positive identification of gene insertion and green fluorescent protein fusion. Biotechniques.

[39] Chen M., Elder R.T., Yu M., O’Gorman M.G., Selig L., Benarous R., Yamamoto A., Zhao Y. (1999). Mutational analysis of Vpr-induced G2 arrest, nuclear localization, and cell death in fission yeast. J. Virol..

[40] Benko Z., Liang D., Agbottah E., Hou J., Chiu K., Yu M., Innis S., Reed P., Kabat W., Elder R.T. (2004). Anti-Vpr activity of a yeast chaperone protein. J. Virol..

[41] Wire M.B., Shelton M.J., Studenberg S. (2006). Fosamprenavir: Clinical pharmacokinetics and drug interactions of the amprenavir prodrug. Clin. Pharm..

[42] Becker Y. (2004). HIV-1 induced AIDS is an allergy and the allergen is the Shed gp120--a review, hypothesis, and implications. Virus Genes.

[43] Furfine E.S., Baker C.T., Hale M.R., Reynolds D.J., Salisbury J.A., Searle A.D., Studenberg S.D., Todd D., Tung R.D., Spaltenstein A. (2004). Preclinical pharmacology and pharmacokinetics of GW433908, a water-soluble prodrug of the human immunodeficiency virus protease inhibitor amprenavir. Antimicrob. Agents Chemother..

[44] Pas T., Bergonzi A., Lescrinier E., Vergauwen B., Van den Mooter G. (2019). Drug-carrier binding and enzymatic carrier digestion in amorphous solid dispersions containing proteins as carrier. Int. J. Pharm..

[45] Griffin L., Annaert P., Brouwer K.L. (2011). Influence of drug transport proteins on the pharmacokinetics and drug interactions of HIV protease inhibitors. J. Pharm. Sci..

[46] Mathias A.A., German P., Murray B.P., Wei L., Jain A., West S., Warren D., Hui J., Kearney B.P. (2010). Pharmacokinetics and pharmacodynamics of GS-9350: A novel pharmacokinetic enhancer without anti-HIV activity. Clin. Pharmacol. Ther..

[47] McCoy C., Badowski M., Sherman E., Crutchley R., Smith E., Chastain D.B., Society of Infectious Diseases P. (2018). Strength in amalgamation: Newer combination agents for HIV and implications for practice. Pharmacotherapy.

[48] Lamb D.C., Maspahy S., Kelly D.E., Manning N.J., Geber A., Bennett J.E., Kelly S.L. (1999). Purification, reconstitution, and inhibition of cytochrome P-450 sterol delta22-desaturase from the pathogenic fungus Candida glabrata. Antimicrob. Agents Chemother..

[49] Durairaj P., Fan L., Du W., Ahmad S., Mebrahtu D., Sharma S., Ashraf R.A., Liu J., Liu Q., Bureik M. (2019). Functional expression and activity screening of all human cytochrome P450 enzymes in fission yeast. FEBS Lett..

[50] Van Leeuwen J.S., Vermeulen N.P., Chris Vos J. (2012). Yeast as a humanized model organism for biotransformation-related toxicity. Curr. Drug Metab..

[51] Cheng J., Wan D.F., Gu J.R., Gong Y., Yang S.L., Hao D.C., Yang L. (2006). Establishment of a yeast system that stably expresses human cytochrome P450 reductase: Application for the study of drug metabolism of cytochrome P450s in vitro. Protein Expr. Purif..

[52] Yan Q., Machalz D., Zollner A., Sorensen E.J., Wolber G., Bureik M. (2017). Efficient substrate screening and inhibitor testing of human CYP4Z1 using permeabilized recombinant fission yeast. Biochem. Pharmacol..

[53] Ali A., Reddy G.S., Nalam M.N., Anjum S.G., Cao H., Schiffer C.A., Rana T.M. (2010). Structure-based design, synthesis, and structure-activity relationship studies of HIV-1 protease inhibitors incorporating phenyloxazolidinones. J. Med. Chem..

[54] Molina J.M., Cohen C., Katlama C., Grinsztejn B., Timerman A., Pedro Rde J., Vangeneugden T., Miralles D., Meyer S.D., Parys W. (2007). Safety and efficacy of darunavir (TMC114) with low-dose ritonavir in treatment-experienced patients: 24-week results of POWER 3. JAIDS J. Acquir. Immune Defic. Syndr..

[55] Zhao Y., Hopkins K.M., Lieberman H.B. (1993). A method for the preparation and storage of frozen, competent *Schizosaccharomyces pombe* spheroplasts. Biotechniques.

[56] Flor-Parra I., Zhurinsky J., Bernal M., Gallardo P., Daga R.R. (2014). A Lallzyme MMX-based rapid method for fission yeast protoplast preparation. Yeast.

[57] Iwaki T., Giga-Hama Y., Takegawa K. (2006). A survey of all 11 ABC transporters in fission yeast: Two novel ABC transporters are required for red pigment accumulation in a *Schizosaccharomyces pombe* adenine biosynthetic mutant. Microbiology.

[58] Kawashima S.A., Takemoto A., Nurse P., Kapoor T.M. (2012). Analyzing fission yeast multidrug resistance mechanisms to develop a genetically tractable model system for chemical biology. Chem. Biol..

[59] Aoi Y., Sato M., Sutani T., Shirahige K., Kapoor T.M., Kawashima S.A. (2014). Dissecting the first and the second meiotic divisions using a marker-less drug-hypersensitive fission yeast. Cell Cycle.

[60] Kurisawa N., Yukawa M., Koshino H., Onodera T., Toda T., Kimura K.I. (2020). Kolavenic acid analog restores growth in HSET-overproducing fission yeast cells and multipolar mitosis in MDA-MB-231 human cells. Bioorg. Med. Chem..

[61] Krohn A., Redshaw S., Ritchie J.C., Graves B.J., Hatada M.H. (1991). Novel binding mode of highly potent HIV-proteinase inhibitors incorporating the (R)-hydroxyethylamine isostere. J. Med. Chem..

[62] Requena D.G.D., Gallego O., Mendoza C.D., Corral A., Jimenez-Nacher I., Soriano V. (2003). Indinavir plasma concentrations and resistance mutations in patients experiencing early virological failure. AIDS Res. Hum. Retrovir..

[63] Lv Z., Chu Y., Wang Y. (2015). HIV protease inhibitors: A review of molecular selectivity and toxicity. Hiv/aids.

[64] Sham H.L., Kempf D.J., Molla A., Marsh K.C., Kumar G.N., Chen C.M., Kati W., Stewart K., Lal R., Hsu A. (1998). ABT-378, a highly potent inhibitor of the human immunodeficiency virus protease. Antimicrob. Agents Chemother..

[65] Becker S. (2003). Atazanavir: Improving the HIV protease inhibitor class. Expert Rev. Anti Infect. Ther..

[66] Larder B.A., Hertogs K., Bloor S., Van den Eynde C.H., DeCian W., Wang Y., Freimuth W.W., Tarpley G. (2000). Tipranavir inhibits broadly protease inhibitor-resistant HIV-1 clinical samples. AIDS.

[67] Cvetkovic R.S., Goa K.L. (2003). Lopinavir/ritonavir: A review of its use in the management of HIV infection. Drugs.

[68] Shah B.M., Schafer J.J., Priano J., Squires K.E. (2013). Cobicistat: A new boost for the treatment of human immunodeficiency virus infection. Pharmacotherapy.

[69] Perez M.A., Fernandes P.A., Ramos M.J. (2010). Substrate recognition in HIV-1 protease: A computational study. J. Phys. Chem. B.

[70] Benko Z., Elder R.T., Liang D., Zhao R.Y. (2010). Fission yeast as a HTS platform for molecular probes of HIV-1 Vpr-induced cell death. Int. J. High Throughput Screen.

[71] Zhao R.Y. (2017). Yeast for virus research. Microb. Cell.

[72] Zhao Y., Lieberman H.B. (1995). *Schizosaccharomyces pombe*: A model for molecular studies of eukaryotic genes. DNA Cell Biol..

[73] Li G., Zhao R.Y. (2018). Molecular cloning and characterization of small viral genome in fission yeast. Methods Mol. Biol..

[74] Agniswamy J., Shen C.H., Wang Y.F., Ghosh A.K., Rao K.V., Xu C.X., Sayer J.M., Louis J.M., Weber I.T. (2013). Extreme multidrug resistant HIV-1 protease with 20 mutations is resistant to novel protease inhibitors with P1′-pyrrolidinone or P2-tris-tetrahydrofuran. J. Med. Chem..

[75] Koh Y., Amano M., Towata T., Danish M., Leshchenko-Yashchuk S., Das D., Nakayama M., Tojo Y., Ghosh A.K., Mitsuya H. (2010). In vitro selection of highly darunavir-resistant and replication-competent HIV-1 variants by using a mixture of clinical HIV-1 isolates resistant to multiple conventional protease inhibitors. J. Virol..

[76] Chetty S., Bhakat S., Martin A.J., Soliman M.E. (2016). Multi-drug resistance profile of PR20 HIV-1 protease is attributed to distorted conformational and drug binding landscape: Molecular dynamics insights. J. Biomol. Struct. Dyn..

[77] Elder R.T., Yu M., Chen M., Edelson S., Zhao Y. (2000). Cell cycle G2 arrest induced by HIV-1 Vpr in fission yeast (*Schizosaccharomyces pombe*) is independent of cell death and early genes in the DNA damage checkpoint. Viral Res..

[78] Benko Z., Zhao R.Y. (2011). Zeocin for selection of bleMX6 resistance in fission yeast. Biotechniques.

[79] Basi G., Schmid E., Maundrell K. (1993). TATA box mutations in the *Schizosaccharomyces pombe* nmt1 promoter affect transcription efficiency but not the transcription start point or thiamine repressibility. Gene.

[80] Zhao Y., Cao J., O’Gorman M.R., Yu M., Yogev R. (1996). Effect of human immunodeficiency virus type 1 protein R (vpr) gene expression on basic cellular function of fission yeast *Schizosaccharomyces pombe*. J. Virol..

